# Supplementary material for: The discovery of novel noncoding RNAs in 50 bacterial genomes
Source: Nucleic Acids Res. 2024 Apr 22;52(9):5152–65. doi: 10.1093/nar/gkae248 (PMC11109978; doi:10.1093/nar/gkae248)
Supplement: gkae248_Supplemental_Files [file gkae248_supplemental_files.zip › Narunsky 50 Genomes RNA Motifs Files.zip!/Publication/61-Geobacter_sp_M21/Figures/LRC-61-29-NC_012918_1_IGR_105_2741637_2741872.sto.pdf]

LRC-61-29-NC_012918_1_IGR_105_2741637_2741872.sto.r2r

5′-○○○○○○○○○○○○○○○○○○○○○○○○○○○○UUYGRGGYYYUGRYRGG●AUUUYY●●GC●CCUC●CCCCC●●UY●GCUCCA●●GGCGCAC●GYCAUCGGR●●●UGRGC●CC●●RG○○○○○G●AUAUUUAR●RYY●●YYAAUY●UCAA●●C●G●○RYG●●●UUUU●YGCUR●GGGGGUR●GRUYUU●GCC●YAA●GUCCGGCC●○○○●●CGGUCGGUCCYUARGGCGCUUU●○
